# Is the Role of Ideologists Central in Terrorist Networks? A Social Network Analysis of Indonesian Terrorist Groups

**DOI:** 10.3389/fpsyg.2020.00333

**Published:** 2020-03-03

**Authors:** Mirra Noor Milla, Joevarian Hudiyana, Wahyu Cahyono, Hamdi Muluk

**Affiliations:** Faculty of Psychology, Universitas Indonesia, Depok, Indonesia

**Keywords:** relational trust, terrorist group, jihadist group, social network analysis, strong ties

## Abstract

This study aims to describe how group leaders operate with their social ties of jihadi terrorists, using social network analysis. Data was collected through documents and interviews from terrorist detainees who were involved in jihadi terrorism activities in Indonesia. We found that relational trust with operational leaders plays an important role in terrorist social networks. More specifically, operational leaders possess a higher degree of centrality and betweenness centrality compared to ideological leaders, as operational leaders happened to possess stronger social ties (with close friends or respected authorities). Furthermore, we also found that terrorist networks in Indonesia consist of a large group of cells with low density, where members are not strongly connected to each other. The only bridges that were strong in these social networks were those involving operational leaders. This study confirmed previous studies that terrorist groups operate in a cell system, but lead to a novel finding that ideological leaders may play a limited or indirect influence in operational networks.

## Introduction

Ever since the September 11, 2001 terrorist attack, the topic of radicalism and terrorism flooded the scientific literature of social science. As this article is written, online database searches (Google scholar) for the keywords ‘terrorism’ and ‘radicalism’ published between 2001 and 2019 resulted in a total of 715,400 articles. This number was almost four times higher compared to the number of articles published between 1980 and 2000 with the same keywords (we found only a total of 187,900 articles in 1980–2000). As a result, we have now retained several scientific models that explain the potential causes of radicalism and terrorism (Gøtzsche-Astrup, 2018). Previous empirical works noted that perceived political injustice (Borum, 2003; Sageman, 2004; Moghaddam, 2005; Della Porta, 2013), social identity consolidation (Kruglanski et al., 2014; Hafez and Mullins, 2015; Sageman, 2017; Newson et al., 2018), personal uncertainty (Wiktorowicz, 2005), revenge (McCauley and Moskalenko, 2011), and need deprivation (Borum, 2003; Silber et al., 2007; Kruglanski et al., 2014) can explain why people engage in the various stages of radicalism.

More recently, Kruglanski et al. (2019) developed an integrative model where ideological narratives and social networks, along with personal needs may interact together in predicting radicalism and terrorism. “Personal needs,” in this context, refers to the need to belong – to live a significant life as a member of the community or society. To live a meaningful life becomes an important goal when the feeling of significance is lacking, such as when people experience personal failure, rejection, and humiliation. Without exposure to an ideological narrative, there would be no cause and therefore violent actions would have no meaning. Similarly, without involvement in social networks, ideological commitment may not easily manifest into real action. Finally, without the burning desire from group members to advance the group’s goal, members of the terrorist cells will not be ready to sacrifice themselves for the sake of the group. Despite the realization of these intertwining factors, one question remains. How strongly must ideological narratives be in the group members’ minds before they are ready to commit acts of self-sacrifice, such as suicide bombings?

The role of ideology in terrorism has been the subject of controversy in the literature. Some argued that religious ideology is the main determinants of terrorism (Harris, 2005; Rausch, 2015; Dawkins, 2016). Others thought that ideology may serve only as a justification of other primary motivations, such as individual needs (Pyszczynski et al., 2003; Webber et al., 2018), economic and political problems (Piazza, 2017; Laqueur, 2017; Anderton and Carter, 2019), or symbolic intergroup conflict (Whitehouse et al., 2019). Despite the apparent disagreement, the current literature lacks firm evidence that demonstrates whether ideology serves as the main determinant or as a justification (Gøtzsche-Astrup, 2018). Moreover, there are only a few research investigations that directly examine the role of ideological narratives in actual terrorist groups. Thus, more research is needed in determining the role of ideology in actual terrorist groups. The present study aims to explore whether ideological narratives are central in terrorist group operations, using the data from actual terrorist groups.

Meanwhile, analysts are still in the dark when trying to determine the nature of terrorist organizations. We need more insights into how the organizations are structured and how these organizations survive, adapt or metamorphose (Jackson, 2006). A significant body of evidence has focused on the advantages of networked and flexible organizations for terrorists compared to more traditional hierarchical organizations (Arquilla and Ronfeldt, 2001; Koschade, 2007). The present study aims to explore potential ways of how terrorist groups operate. Considering that terrorist groups are formed through strong social bonds such as kinship (Sageman, 2004, 2011) and friendship (Milla and Hudiyana, 2019), we are questioning whether ideological narratives are central in terrorist group operations.

As described by Yuki (2003), people in Western cultures tend to emphasize the categorical distinction between ingroups and outgroups, while East Asians may have a stronger tendency to think about groups as predominantly relationship-based. We argue that within a communal society such as Indonesia, ideological narratives may play a less important role for members/followers compared to the relational trust and fulfillment of personal needs. For Indonesian terrorist groups, leaders who inspire members may play a more significant role in obtaining devotion from their members, compared to the ideological narratives itself. As a country which adopts Asian culture, Indonesia is a society with a large gap of power between leaders and members (Hofstede and Bond, 1984; Irawanto, 2009), and a society where harmony, loyalty, and compliance inside community is paramount (Triandis et al., 1986; Rahardjo, 1994; Rajiani and Jumbri, 2011). These cultural practices may shape how the group operates in order to advance their goals, including terrorist groups. Consequently, there may be a lesser need for leaders to inspire through ideological indoctrination. Rather, leaders should inspire the loyalty of members through their discipline, benevolence, and relational ties (Liu, 2015). Such leaders should possess a more central role in the group compared to ideological leaders or experts. Here, ideological narratives do not serve as a single underlying cause of radicalism. However, relational ties with an operational leader within a terrorist cell determine its members’ willingness to commit violent behavior.

### Indonesian Terrorist Groups Dynamics: How Leaders Manage Their Members

In general, Sageman (2004) explained that the formation of religiously motivated terrorist groups begins with people who decided to join small groups. Within these groups, the people live together for a certain period of time where they intensely discuss topics of religious ideology. Some of these individuals then joined jihadi military training (in Afghanistan, for example) and this further strengthens the group identity and ideological commitment (Milla et al., 2013, 2019). In this stage, the individuals belong to a very small group with high relational bonds. Sageman (2004) explained that these small groups consist of people whose friendships have developed intensely and individuals possess similar backgrounds. Thus, intense bonds can be found in such cliques. In other words, terrorist cells usually consist of small groups with strong ties where personal relationships are significant to each member.

Previous works have pointed out the role of ideological narratives as a determinant in explaining terrorism (Crenshaw, 1985). However, whether ideological narratives play a paramount role in the formation of terrorist cells has never been explained. Ideology is believed to inspire only when it is spread within the collectively shared reality (Hardin and Higgins, 1996; Kruglanski et al., 2013). This means that social ties are initially formed without strong ideological propaganda and such narratives are emphasized only after group commitment is established. At the formative stage, leaders or mentors play a more important role, especially in maintaining compliance and loyalty to the group (Milla et al., 2013). The leadership position is essential in strengthening the ideologization process through exclusivity and isolation, and encourage commitment to the point of no return (Milla and Umam, 2019). These leaders inspire loyalty as well as ensuring the fulfillment of members’ personal and psychological needs.

The centrality of this leadership role explains why terrorist cell groups are less ideological and more relational. These leaders are not the main ideologists where religious inspiration and *fatwa* (preaching) are central for members’ devotion. Rather, they utilize religion and ideology mainly as the source of justification. For instance, a member may seek revenge on an outgroup or the government, and it is the job of these leaders to provide religious verses that may justify such revenge. This implies that ideology can strongly motivate in a context where it is in line with personal needs (Kruglanski et al., 2019). However, whenever the ideological propaganda is out of touch with personal needs, the motivation will not be as powerful. This is why relational bonds are important since such bonds will ensure trust. The operational leaders, who also happen to be mentors, have strong interpersonal ties with their followers (Milla et al., 2013). This phenomenon is explained by Sageman as a leaderless jihad (Sageman, 2011), but they are actually not leaderless. The leaders are simply not ideological leaders. Rather, they act more as the operational leaders who organize the recruitment of members, manage the group, and orchestrate the terrorist actions. Thus, these leaders may satisfy the needs of members by providing mutual relationships, inspiring devotion, exerting benevolence, or setting an example through personal integrity.

Within the terrorist groups, there are usually people who are respected as ideologists or preachers. They are known as leaders who are prophetic, possess a high level of religious knowledge, are looked up to in terms of morality, and their preachings should always be obeyed. In spite of this, they are less likely to experience direct contact with their members. One of the most famous figures to play a role of an ideological leader is Abu Bakar Ba’asyir, whose teachings inspired the terrorist networks but he was not directly involved in the execution of the terrorist actions (Atran, 2006). These preachers are only responsible to provide the group with profound ideological knowledge. However, since such profound knowledge relies on higher-order thinking and deeper cognitive understanding, its attractiveness for followers is limited (Magolda, 2008). In addition, such profound knowledge may inspire agency and autonomy, where group dogmas and doctrines may be questioned by individuals (Magolda, 2008; Harari, 2016). That sense of agency and autonomy may endanger the cohesiveness and unity of terrorist groups. Previous works demonstrated that the support for group hierarchy and social dominance – common in terrorist networks – are robust for those who tend to be closed-minded (Onraet et al., 2011). In addition, individuals who are prone to heuristic biases (as opposed to critical thinkers) are more likely to be recruited into terrorist organizations (Milla, 2005). Consequently, ideological leaders may merely satisfy the needs for certainty and order (Jost et al., 2008a; Hogg, 2014).

An ultra-conservative group such as the terrorist group provides system-justifying belief systems which rationalize the social and political arrangements they deemed as necessary (Jost et al., 2008b). Such group serves to maintain the members’ personal satisfaction – a palliative function – in order for them to cope with reality (Jost and Hunyady, 2003). There are three ways that group can provide system-justification needs (Jost et al., 2008a): (1) through offering cognitive order and certainty – the epistemic needs; (2) through reducing the threat and stress – the existential needs; and (3) through providing a shared reality and the needed relationship - the relational needs. Within the terrorist groups, it is possible that ideologists may be more adept in providing cognitive certainty through their preachings while operational leaders, who interact intensely with the members may provide the members with existential and relational needs.

Group leaders may also inspire trust through exhibiting one or more of the three trustworthiness styles (Mayer et al., 1995). First, they can show their ability to the members so that these members perceive them as competent. Second, they can provide kindness and supportive attitudes in various behavior so that the members perceive them as benevolent. Third, they can guide the members through integrity and so the leaders may be perceived as a role model or someone to look up to. Such trustworthiness is paramount in order to enhance members’ satisfaction (Gilstrap and Collins, 2012), members’ performance (Hakimi et al., 2010) as well as increasing outcome favorability (Lin et al., 2009).

We assume that the operational leaders’ role is more central in maintaining group continuity, compared to ideological actors. The rationale for this assumption is that strong leader–follower interactions can provide trustworthiness as well as ideological justifications for the members’ system-justifying needs. In the formation of group commitment, the leaders’ strategy in ensuring the fulfillment of followers’ personal needs is paramount. Further ideological knowledge may be less important, and so the trust toward ideological actors is not necessary.

### Relational Dynamics in Terrorist Groups: The Role of the Indonesian Cultural Context

Strong and stable social relations promote a sense of security within collective entities (Yamagishi et al., 1998). This relational issue should be a concern in discussing the group in a collective culture and communal society. In a communal society such as Asia, people often emphasize the role of relational hierarchies (Liu, 2015). Extreme power gaps may not be seen as anti-democratic or authoritarian, but rather as a necessary structure to maintain order and harmony. In such a context, the leader–follower relationship may not be manifested in the transactional benefits of each party involved. Rather, it is manifested in devotion and loyalty to authority, where the leaders are solely responsible for ensuring the fulfillment of the members’ well-being (Liu et al., 2010). As an Asian society, Indonesia may culturally share such tendencies.

Loyalty and devotion to authority may be the most apparent moral values in the Indonesian communal culture. Such values are shared in many Asian philosophies, such as Confucian thoughts (Rosemont, 2015). People who do not show loyalty or devotion to a group are seen as those who can ruin harmony and order. Devotion to authority figures, such as the elderly or assigned community leaders, has been internalized ever since individuals interact with their most immediate authority, that is, their parents. It is not surprising that such virtues are practiced in the context of terrorist groups. For those within terrorist groups, the meaningful interactions may be less transactional and equal, but rather hierarchical. Complete obedience to authority may not be seen in a negative light but is seen favorably. Consequently, once the leaders have set the moral grounds, it is easier to manage the members’ loyalty, even without ideological inspiration (Milla et al., 2019). This also implies a shift of responsibility, because the members may perceive that the responsibility of actions completely belong to authority figures whom they trust (Bandura et al., 1996; Milla, 2010). Furthermore, whatever indoctrinations and moral justifications of violence that the leaders propose, the members would be willing to commit as long as they trust the leaders. Thus, the leaders may need to show personal integrity through inspiring devotion.

Yuki’s (2003) framework proposes that Asian collectivism is important to maintain relational harmony, especially within groups where members are fully devoted to the respected figures, such as group leaders and key figures in society. Trust occurs only in the group with strong social cohesion. In order to create such strong cohesion, it is important for members to completely obey the authority figures. In terms of in-group cooperation and coordination, relational trust is more important than the narratives of ideology provided inside the groups. Relational trust, as explained by Bryk and Schneider (2002), is rooted in a complex cognitive activity of discerning the intentions of others that occur within a set of interpersonal relationships and are formed both by the group structure and by the particularities of an individual in the group, localized in its own culture, history, and local understandings.

For Indonesian people, personal needs may also be less individualistic (e.g., personal achievement). For instance, motivation to be a hero, martyr, or to experience sensations is one of the several personal motivations for individuals to join terrorist organizations (Kruglanski et al., 2014). Such self-enhanced motivation may not be shared by Indonesian terrorist group members. Rather, the motivation may be more relational, such as to make the authorities proud and to avoid strife within a group, or to maintain harmony. Previous work has demonstrated that individualistic cultures, such as Western societies, may promote self-enhanced motivation as a primary orientation, while collectivistic cultures, such as Asia, may be oriented toward avoidance of relational loss and harmony-seeking (Elliot et al., 2001).

In the present study, we assume that the leaders’ role is central in terrorist networks because they are the central decision-makers who inspire loyalty and devotion from their followers. In addition, followers in Indonesian terrorist groups may be less inclined to be inspired by individual ideological understanding or heroic motivations. The centrality of social networks of terrorist organizations may be shaped by operational rather than ideological leaders. Therefore, this study aims to show that operational leaders have a significant role in terrorist networks by establishing relational trust with their members.

## Materials and Methods

### Research Design

This study uses social network analysis to examine the network of militant Islamic groups in Indonesia. This approach aims to understand exactly how groups of individuals interact and operate, and consequently, how they behave. This is a methodology that fuses mathematics, anthropology, psychology, and sociology. A “social network” is a social structure made up of individuals (or organizations) called “nodes,” which are tied (connected) by one or more specific types of interdependency, such as friendship, kinship, common interest, financial exchange, dislike, sexual relationships, or relationships of beliefs, knowledge or prestige (Passmore, 2011). Social network analysis allows us to map and measure complex, and sometimes covert, human groups and organizations. The method focuses on uncovering the pattern of people’s interaction. Social network analysis provides a powerful way of structuring knowledge about the relationship between concepts and people (Koschade, 2007). By using the framework of social network analysis, we were able to detect the ‘stars’ or ‘well-connected figures’ of the networks by computing the number of connections a person has with other people in the networks and compare it with overall connections (Scott, 1988). In addition, we also attempted to triangulate the findings by obtaining qualitative data from documents and interviews.

### Data Collection

Ethical approval was not required for this study in accordance with the national and institutional requirements. However, the proposal was first examined by the university officials and inter-university evaluators before we were granted permission to execute the study. The evaluators and officials were Professors with expertise in militant extremism who came from various universities in Indonesia and Germany. The universities were Jacobs University Bremen – Germany, Sultan Syarif Kasim State Islamic University of Riau – Indonesia, Universitas Indonesia – Indonesia, and Universitas Riau – Indonesia. They examined our research questions, interview guidelines, and technical issues (e.g., ethical consent) in compliance with Indonesian laws. We were allowed by the prison officials, in cooperation with Badan Nasional Penanggulangan Terorisme (Indonesian National Agency for Combating Terrorism), to interview the detainees in prisons who were involved in terrorism. These detainees were willing to be interviewed, but the consent was managed by the prison officials since they were the ones who directly asked for their consent. There were some detainees who were unwilling to be interviewed for various reasons, so we did not interview these unwilling participants. We entered a room in the prisons, where the officials have already brought the detainees who are willing to participate.

The data were collected from documents and interviews. The documents consisted of articles that described the terrorist leaders, written testimonies from terrorists in the prisons, and published biographies of the terrorist leaders. Examples of the documents that we analyzed were published articles entitled “*Indonesia Backgrounder: How The Jemaah Islamiyah Terrorist Network Operates*” which was published in December 11, 2002; “*Terrorism in Indonesia: Noordin’s Networks*” which was published in May 5, 2006; and “*Indonesia: Jihadi Surprise in Aceh*” which was published in April 20, 2010. Other documents were written diaries of terrorist detainees in prisons and a published biography of the terrorist leaders such as a book entitled “*Membongkar Jamaah Islamiyah*” or “Unveiling the Jamaah Islamiyah” published in 2007. From these documents, we obtained information about the roles of actors within the network, as well as their interpersonal relations which contributed to the formation of the network. We have also gained a deeper understanding of how members of the terrorist groups are connected through kinship, marriage and friendship (Sageman, 2004).

Meanwhile, the interviews were conducted across the six terrorist groups. The six terrorist organizations that we analyzed were identified as Bali Bombing Group I, Bali Bombing Group II, J. W. Marriott Bombing Group, Aceh Group, Poso Group, Solo Group, and KOMPAK Group. More specifically, we collected the information from members of several Islamist militant organizations who resided in two Indonesian prisons (Jakarta and Cilacap prisons), as well as information from known members of Islamist militant organizations in three Indonesian cities (Jakarta, Pekanbaru, Lamongan) outside prisons. We interviewed a total of 18 terrorist detainees. We asked the interviewees to list the names of the terrorist actors (leaders and members) inside and outside their organizations. Additionally, we also probed them regarding their relationship with the actors they have mentioned. The interview questions were focused on the questions related to the relationship and interaction between members of their groups, between members and their leaders, as well as between the group leaders. The informants were selected from groups that were involved in certain terrorist activities, such as jihad mobilization in Indonesia.

We successfully obtained 163 nodes and 888 directed ties (edges) across six terrorist organizations. Among these nodes, three nodes were ideological leaders and six nodes were operational leaders. We explained the details of these nodes in the following section.

### Defining Terrorist Group Leaders

Operational leaders were defined as individuals who organized recruitment, manage the group, and orchestrated the terrorist actions. The operational leaders are abbreviated as OL1, OL2, OL3, OL4, OL5, and OL6. Meanwhile, ideological leaders were defined as individuals who assume a leadership role in ideological propaganda. They orchestrated the moral disengagement narratives and provided the verses of the holy text to justify violence and terrorism. They were known as preachers, who possess profound religious knowledge and are regarded as *ulama* (Imam of Muslims). The ideological leaders are abbreviated as IL1, IL2, and IL3.

Generally, operational leaders (OL1, OL2, OL3, OL4, OL5, and OL6) were either influential figures in their communities or people whose charisma was so profound that it could inspire devotion, especially in young males. Several of these operational leaders were highly educated, as some of them graduated from reputable universities in Indonesia and Malaysia. They were usually skilled in technical skills such as computer programming or chemistry (for the creation of explosives).

Meanwhile, ideological leaders (IL1, IL2, and IL3) were known to possess the sheer intellectual capacity and profound scholarly knowledge of religion. Within the terrorist network, they were regarded as great Imams whose preaching is central in the recruitment process and prepared the young members to commit self-sacrifice. Additionally, they provide the ideological justifications in practically any members’ activities. No members of the group, as well as the operational leaders, dared to question the ideological leaders. Based on our classification, individuals were categorized either into ideological leaders or operational leaders exclusively. The individuals could play either of the roles, but in our research, there are no leaders who have both roles at the same time.

### Strategy of Analysis

Data obtained from the documentation and interviews were coded. To describe the role of each leader, we used verbatim data obtained from various documents. We analyzed the verbatims by using thematic analysis. We categorized the personal style of leadership adopted by the ideological and operational leaders by using the Three System Justification Needs Framework (Jost et al., 2008a; Hennes et al., 2012) and the Three Styles of Organizational Trust (Mayer et al., 1995).

According to The Three System Justification Needs Framework, there are three types of system justifying needs: (1) Epistemic needs, that is the need for cognitive certainty and order, (2) Existential needs, that is the need to avoid existential threats and to reduce distress, and (3) Relational needs, that is the need to establish social relations and a shared reality with social networks. We analyzed the data by classifying each leader based on the type of needs they provided to their followers. Furthermore, according to Mayer et al. (1995), there are three types of trustworthiness: (1) Ability, which refers to the trustworthiness based on the relevant skills and competences, (2) Benevolence, which refers to the trustworthiness based on the willingness to help and support the truster, and (3) Integrity, which refers to the trustworthiness based on the consistency of adhering to the accepted principles. These two theoretical frameworks are appropriately used in this context because the identification of these categories is meaningful and consistent with our proposed explanation regarding why non-ideological needs may be more important in establishing stronger relational ties within a terrorist group.

The first step in any attempt to analyze a social network is to construct a contextual background of the relationships between nodes (the type of relationships and the degree of relationship quality). This relational background must be accomplished in order to understand the physical environment of the network. The relations or ties between each node were weighted based on relational trust, taking into account the closeness, roles, and frequency of interactions. However, we analyzed the in-degree and out-degree scores to determine the number of relationships that each of the nodes possesses. Furthermore, the data was organized by patterns of interaction and activity between members of the network cell. Based on the patterns of interaction of each member, the data were coded and analyzed by the Gephi software. All data that has been collected are categorized for actors based on their role and their relations in the networks. The analyzed data is available in Supplementary Data Sheets 2, 3.

We then computed the scores of overall network density as well as centrality (degree, in-degree, out-degree, and betweenness) for each leader node in the network, using the Gephi software. Degree centrality scores indicate the overall well-connectedness of each node – how many edges or ties that each node has compared to overall ties inside the networks; while in-degree centrality indicated the direct relationship of other nodes with the respective node – how many nodes interacted with a certain single node (Scott, 1988). In contrast, out-degree centrality scores refer to the direct relationship of a single node to other nodes – the total number of interactions from a single node directed to other nodes (Scott, 1988). Finally, betweenness centrality scores indicate the node’s ability to bridge other nodes – how many other nodes are linked by a single node as a bridge (Freeman et al., 1979). A score closer to ‘1’ represents a stronger in-degree, out-degree, and betweenness centrality while a score closer to ‘0’ represents a weaker in-degree, out-degree, and betweenness centrality.

Other than the scores of centrality, we also computed the scores of modularity, density, and path diameter. Modularity refers to an estimate of divisions inside a network. A value of more than 0.5 indicates that there are clear divisions inside of a network. Meanwhile, density is an estimate of the proportion of the relationship in the network to the total number of possible relationships. A value closer to ‘1’ indicated a big network where each member is associated with other members, while a value closer to ‘0’ indicated a collection of separate networks within a network where many members do not communicate with other members inside the network. Finally, network diameter refers to the estimate of the longest distance of travel between members inside the network.

## Results

### Characteristics of Leader Nodes: The Distinctive Role of Operational Leaders and Ideological Leaders

In this section, we summarize the characteristics of both ideological and operational leaders based on the documents and interviews that we obtained. We analyzed the data from interviews and documents by categorizing the needs provided by the figures inside the system or group in the framework of System Justification Theory (Jost et al., 2008a) while referring to the three types of trustworthiness (ability, benevolence, integrity). These two theoretical frameworks are appropriately used in this context because the identification of these categories is meaningful and consistent with our proposed explanation regarding why non-ideological needs may be more important in establishing stronger relational ties within a terrorist group. See Table 1 for a complete thematic analysis.

**TABLE 1:** Characteristics of leaders (summarized from documents).

**#**	**Initials (Roles)**	**Style of Trustworthiness (Mayer et al., 1995)**	**The Needs Provided to Members (Jost et al., 2008a)**
1	IL1 (Ideological leader)	Ability – trustworthy because of the profound knowledge in religion	Epistemic needs – satisfies the needs of order and certainty
2	IL2 (Ideological leader)	Ability – trustworthy because of the profound knowledge in religion	Epistemic needs – satisfies the needs of order and certainty
3	IL3 (Ideological leader)	Ability – trustworthy because of the profound knowledge in religion	Epistemic needs – satisfies the needs of order and certainty
4	OL1 (Operational leader)	Benevolence – trustworthy because the leader provides resources and material needs	Existential needs – satisfies the needs to reduce stress
5	OL2 (Operational leader)	Integrity – trustworthy because the leader values the group norms and inspires discipline	Relational needs – satisfies the need for devotion
6	OL3 (Operational leader)	Benevolence – trustworthy because the leader inspires loyalty as a good friend	Relational needs – satisfies the needs of social relationship
7	OL4 (Operational leader)	Integrity – trustworthy because the leader values the group norms and inspires discipline	Relational needs – satisfies the need for devotion
8	OL5 (Operational leader)	Benevolence – trustworthy because the leader provides a paternalistic figure	Relational needs – satisfies the need for devotion
9	OL6 (Operational leader)	Integrity – trustworthy because the leader values the group norms and inspires discipline	Relational needs – satisfies the need for devotion

Compared to the three ideological leaders, the six operational leaders tend to be more relational in their interactions with their followers. Operational leaders such as OL1, OL2, OL4, OL5, and OL6 tend to exert the characteristics that inspire loyalty, obedience, and may provide the personal needs (e.g., economic needs and existential needs) of followers as well. For instance, the followers of OL5 saw him as a paternalistic figure who was very fatherly and supportive of his followers. Similarly, OL1 was perceived as a very accommodative and resourceful figure, who can manage the followers’ personal needs rather easily. OL1 could easily provide the economic needs of his followers, such as halal goods (as opposed to forbidden goods, usually bought in general markets). Meanwhile, the followers of OL2 and OL6 tend to perceive him as a powerful and charismatic figure whose teachings must be obeyed, as he may exert his influence through fear. This is similar to OL4, whose followers perceived him to be strict and fierce.

However, other operational leaders, such as OL3, tend to secure devotion because of his meaningful relationships with his followers, rather than because of his inspiring characteristics or powerful attitudes. For instance, one of the followers of OL3 devoted his life to commit actions of self-sacrifice because he felt that OL3 was personally close to him; a best friend and a part of his family. He was willing to risk his life for terrorist activities led by OL3 because he was his childhood friend.

This was in contrast with ideological leaders, where the relational approach and meaningful interactions were not emphasized. Rather, ideological leaders merely provide moral justifications for violent actions, determine the readiness of members in the actions (which can be done indirectly), as well as implanting the indoctrination of terrorism in the followers’ minds. These narratives often were only powerful when individual commitment to the group has been established. Further, the indoctrination of religious knowledge may only be relevant when it is in line with their personal needs in a specific context. Thus, this may be less important in the daily group interactions and dynamics within the terrorist group. Verbatim example for the qualitative analysis and short profile for each leader are illustrated in Supplementary Appendices A, B.

### The Overall Network Structure of Islamic Radical Groups in Indonesia

First, we ran the Gephi software to examine the overall network structure and the density of the network. On average, a node had five relationships with other nodes, where the average distance from one node to another node is three steps (path length). The structure of the network is low in density (Density = 0.034), indicating that the relationship between actors are not directly connected with one another but through single connectors who act as intermediaries (bridging ties). We found the score of Average Clustering Coefficient = 0.465, Average Path Length = 3.201, Modularity = 0.549, Average Degree = 5.479, Average Weight Degree = 4.438, and Network Diameter = 7. Taken together, these results indicate that even though a lot of people were involved in a single network, they tend to operate in separate cells, forming various independent groups and were connected to each group only through a single bridge of node.

**FIGURE 1:**
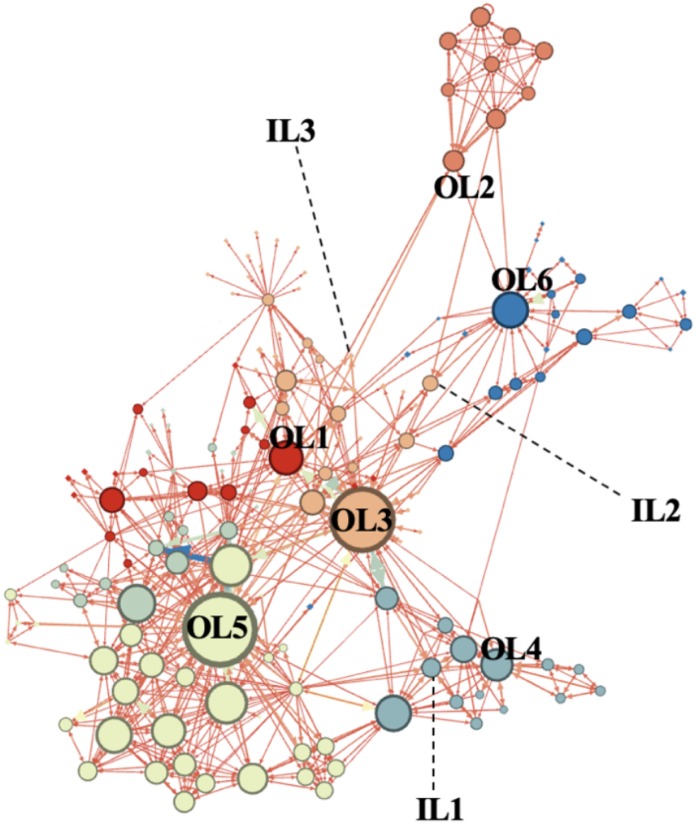
Overall network structure of Indonesian terrorist organizations.

The network structure is illustrated in Figure 1. From the network structure, we can infer that there were at least six groups identified in the network, though all of the nodes formed a single network structure. Interestingly, we found a total of more than six clusters of networks in the structure. This means that there was a group that may not be united as a single cohesive structure. The two nodes with the boldest color (OL3 and OL5) are the nodes that possess the highest betweenness centrality.

### Centrality of Ideological Leaders and Operational Leaders

From Figure 1, we can also observe that operational leaders are more central compared to ideological leaders. We then computed the degree centrality, in-degree centrality, out-degree centrality, and betweenness centrality for each leader. Table 2 illustrates the scores for all centrality indices across all nine leaders. Consistent with our prediction, we found ideological leaders to be less influential, in which all centrality scores were numerically lower compared to the operational leaders. The three highest degree centrality (the number of interactions of other nodes with the reference node) scores were earned by operational leaders (OL3, OL5, and OL6) while the three lowest degree centrality scores were earned by ideological leaders (IL1, IL2, IL3).

**TABLE 2:** Centrality of nine leader nodes.

**#**	**Initials (Roles)**	**Degree Centrality (Weighted)**	**In-Degree Centrality (Weighted)**	**Out-Degree Centrality (Weighted)**	**Betweenness Centrality**
1	IL1 (Ideological leader)	18 (16.2)	9 (8.7)	9 (7.5)	123.5
2	IL2 (Ideological leader)	13 (10.6)	7 (5.9)	6 (4.7)	608.6
3	IL3 (Ideological leader)	2 (2.0)	2 (2.0)	0 (0.0)	0.0
4	OL1 (Operational leader)	32 (29.3)	17 (14.4)	15 (14.9)	1472.8
5	OL2 (Operational leader)	21 (17.2)	10 (8.8)	11 (8.4)	1858.6
6	OL3 (Operational leader)	65 (60.4)	33 (32.1)	32 (28.3)	5078.9
7	OL4 (Operational leader)	31 (25.5)	16 (13.8)	15 (11.7)	1138.9
8	OL5 (Operational leader)	75 (66.9)	38 (37.0)	37 (29.9)	4645.4
9	OL6 (Operational leader)	38 (30.3)	18 (15.3)	20 (15.0)	3172.7

Similarly, the highest in-degree centrality (how many other nodes interacted with a reference node) score was owned by OL5. Again, the highest out-degree centrality (how many other nodes that the reference node was in contact with) score was owned by OL5, although it was only slightly higher compared to OL3. Both were operational leaders. Even though OL2 only scored slightly higher in-degree centrality compared to IL1, the score of betweenness centrality (how likely a node is to bridge other nodes) of OL2 was much higher compared to all ideological leaders. Thus, the results also show that operational leaders were more likely to connect the members with each other within the terrorist networks. One of the ideological leaders, IL3, scored 0.0 in betweenness centrality and out-degree centrality, which shows that IL3 did not interact with other nodes, even though other nodes show that there is a perceived relationship with IL3 (In-degree centrality = 2.0).

## Discussion

The present research argued that terrorist networks in Indonesia will be less influenced by ideological leaders. Rather, the actors who played a central role in terrorist networks are operational leaders. The rationale behind this assumption is that relational trust should be maintained inside the group as a mechanism to bond the individuals inside the group to become highly committed. In this sense, the operational leaders managed to establish relational trust along with relational hierarchy within the group. Ideology, on the other hand, served only as moral justification, especially for preparing individuals to commit self-sacrifice or violence. This can happen indirectly, even without direct contact with the ideological leaders. From the analysis, we found that, indeed, operational leaders were the actors that play a central role in terrorist networks while ideological leaders were less central. Not only that each and every one of the operational leaders possessed higher scores of degree centrality (both in-degree and out-degree), but they also scored higher in betweenness centrality. This means that not only do these operational leaders have the most contact with other group members, but they also linked the followers to each other and with other members of distinct terrorist groups.

The results imply that since operational leaders are the central player in the networks, they need to secure their followers’ loyalty and maintain their followers’ needs by various means. According to the theoretical proposition by Kruglanski et al. (2019), having powerful ideological narratives may not be enough. Members’ personal needs should also be addressed to motivate them into joining the group, continuing their membership, and committing self-sacrifice. Therefore, the leaders’ capability to secure the commitment of their members is paramount. Our qualitative data indicated that operational leaders such as OL1, OL3, and OL5 inspired loyalty because they were either a good friend, assumed a paternalistic role (fatherly figure), or can fulfill personal needs, such as economic needs.

In addition, the cultural context of Indonesia may also explain why such relational emphasis in the network might happen. Our qualitative data indicated that operational leaders such as OL2, OL4, and OL6 exerted fear over their followers. They maintained a relational hierarchy within the group. In a communalistic society such as Indonesia, such a demonstration of power may not be seen as anti-democratic or authoritarian (Liu et al., 2010). Rather, it was reciprocal, in which the leaders are in supreme responsibility to protect the followers and to ensure the security of followers. As an act of reciprocity, the followers completely obey the authority and trust the leaders. Such a phenomenon is not new in Asian culture, where the hierarchical relationalism is paramount in maintaining harmony (Liu, 2015). However, compared to OL3 and OL5, all centrality scores for OL2, OL4, and OL6 tend to be lower. This might indicate that relational trust exhibited by OL3 and OL5 may be more influential to followers than the absolute authoritarian style exhibited by OL2, OL4, and OL6 (see Table 1).

The results that show how ideological actors play a less central role in the network can perhaps be attributed to the proposition by Kruglanski et al. (2019). Without the fulfillment of personal needs, the motivation to be committed in the network may not be quite strong. With this in mind, ideological narratives may be important as long as it is in line with personal needs. For instance, someone whose goal is to avenge the death of his family may join the terrorist group, not because of the divine commandments, but to kill the members of the outgroup. However, it is important to justify such motivation with ideological narratives, to morally disengage from the violence. Here, a deep understanding of religious teachings may not be necessary for members. A deep understanding of ideology may not be useful because such knowledge may be too difficult for all members to comprehend. Additionally, it may be more difficult to maintain loyalty in the context where the members always question the teachings. Thus, deep understanding may be counterproductive for group cohesiveness (Harari, 2016).

Further ideological indoctrinations may be necessary only for justification of self-sacrifice and violence (the final stage of violent extremism) but are not necessary for daily group dynamics (Milla et al., 2013). In daily group processes, the maintenance of individuals’ needs is much more important than having a strong ideological commitment. Such maintenance may enhance group commitment and bolster social identification with the terrorist groups. When commitment is high, it would be much easier to indoctrinate the members.

We also found that the terrorist network in Indonesia was not a dense network since the density score was relatively low. This explained why the networks might be difficult to destroy. Previous work suggests that network density is positively related to the ease of the network authority’s command and control (Granovetter, 1983). It may also render the network more vulnerable as such a network is more likely to fall into rapid deterioration once the key figures are eliminated (Koschade, 2006). As we assumed, the network consisted of a strong bond that was based more on interpersonal relational ties. The strong bond with a group is centered on the central figure rather than the group ties as a whole (Yuki, 2003). This study also confirms previous findings that terrorist networks are often engaged in a cell system (Sageman, 2004; Koschade, 2007; Wheatley, 2007).

The characteristics of terrorist cells are unique, in which the cells are connected by interpersonal relations which act as bridging ties between actors. Again, the operational leaders, serving as actors with high betweenness centrality may play a great part in connecting the cells. The groups were led by the leaders who emphasized relationality and placed less emphasis on ideology. Therefore, this group is more likely to form a cell system, in which the action of each cell is autonomous and very collective on the inside (Matthew and Shambaugh, 2005) under the central role of a leader (Chappel, 2002; Ressler, 2006; McCauley and Moskalenko, 2011). Cell systems, as observed in our results, may actually reduce the chance of detection and allow high flexibility in operation (Stern, 2003; Dishman, 2005; Hoffman, 2006). On the other hand, however, the problem of coordination and control arises mainly due to the lack of trust and coordination between cells (Chappel, 2002; Sageman, 2004; Koschade, 2007). Consequently, some of the cells may seem to adopt different strategies and even became hostile with one and another.

This finding may also challenge previous assertions, such as the notion of a leaderless jihad by Sageman (2011), who described that terrorist groups work in the cell system without a leader. Our network analysis demonstrated that key actors, which possess high degree centrality, were actually leaders. However, they are not ideological leaders, but those who orchestrated the actions, who recruited the followers, and who manage the daily needs of followers. Thus, this may support previous assumptions that there is actually a form of collective leadership inside terrorist groups (Crenshaw, 1985; Arquilla et al., 1999; Friedrich et al., 2009) based on the shared values (Wheatley, 2007).

Although our social network analysis has successfully demonstrated that the role of operational leaders is more central than ideological leaders within the network, social network analysis should not be used to explain causal patterns. So, the results in this study cannot be used to claim the causal effect of the influence of relational trust and ideology in explaining terrorism. Further, the distinction between operational and ideological leaders was obtained through our interviews with the members as well as the documents. We did not conduct a systematic approach to distinguish the two roles and test interrater reliability. We acknowledge these as the limitations of this study.

## Conclusion

In summary, we found that Indonesian terrorist networks consisted of a single network, but with separate cells. The key actors inside the networks were not ideological leaders who assume the role of religious indoctrination. The key actors were operational leaders who recruit and manage the followers as well as preparing these followers to commit self-sacrifice. In order to maintain or increase commitment to these groups, leadership capability and relational factors may play a stronger role compared to ideological narratives.

## Data Availability Statement

The datasets generated for this study are available on request to the corresponding author.

## Ethics Statement

Ethical review and approval was not required for the study on human participants in accordance with the local legislation and institutional requirements. Written informed consent for participation was not required for this study in accordance with the national legislation and the institutional requirements. Written informed consent was obtained from the individual(s) for the publication of any potentially identifiable images or data included in this article.

## Author Contributions

MM was the first author who proposed the theoretical basis, hypotheses, and wrote the Introduction and Discussion (40% contribution). JH was the second author who conducted the analysis of data and helped to writing the Introduction, Materials and Methods, and Discussion (35% contribution). WC was a co-author who contributed to the analysis of data (15% contribution). HM was a co-author who contributed to the theoretical discussions (10% contribution).

## Conflict of Interest

The authors declare that the research was conducted in the absence of any commercial or financial relationships that could be construed as a potential conflict of interest.

## References

[B1] Anderton C. H. Carter J. R. (2019). *Principles of Conflict Economics: The Political Economy of War, Terrorism, Genocide, and Peace.* Cambridge: Cambridge University Press.

[B2] Arquilla J. Ronfeldt D. (2001). *Networks and Netwars: The Future of Terror, Crime, and Militancy.* Santa Monica, CA: Rand Corporation.

[B3] Arquilla J. Ronfeldt D. Michell Z. (1999). “Networks, netwar, and information age terrorism,” in *Countering the New Terrorism*, ed. Lesser I. O. (Santa Monica, CA: RAND Corporation), 39–84.

[B4] Atran S. (2006). The moral logic and growth of suicide terrorism. *Wash. Q.* 29 127–147. 10.1162/wash.2006.29.2.127

[B5] Bandura A. Barbaranelli C. Caprara G. V. Pastorelli C. (1996). Mechanisms of moral disengagement in the exercise of moral agency. *J. Pers. Soc. Psychol.* 71 364–374. 10.1037/0022-3514.71.2.364

[B6] Borum R. (2003). Understanding the terrorist mind-set. *FBI L. Enforcement Bull.* 72:7.

[B7] Bryk A. Schneider B. (2002). *Trust in Schools: A Core Resource for Improvement.* New York, NY: Russell Sage Foundation Available at: www.jstor.org/stable/10.7758/9781610440967 (accessed November 19, 2019).

[B8] Chappel G. G. Jr. (2002). *A Terrorist Organization as a Systems: Unleasing Wardens’s Five Ring Model*. Dissertation (unpublished), The Naval War College Department of Joint Maritime Operations.

[B9] Crenshaw M. (1985). An organizational approach to the analysis of political terrorism. *Orbis-A J. World Affairs* 29 465–489. 10.1385/FSMP:1:3:169 25870041

[B10] Dawkins R. (2016). *The God Delusion.* New York, NY: Random House.

[B11] Della Porta D. (2013). *Clandestine Political Violence.* Cambridge: Cambridge University Press.

[B12] Dishman C. (2005). The leaderless nexus: when crime and terror converge. *Stud. Conflict Terrorism* 28 237–252. 10.1080/10576100590928124

[B13] Elliot A. J. Chirkov V. I. Kim Y. Sheldon K. M. (2001). A cross-cultural analysis of avoidance (relative to approach) personal goals. *Psychol. Sci.* 12 505–510. 10.1111/1467-9280.00393 11760139

[B14] Freeman L. C. Roeder D. Mulholland R. R. (1979). Centrality in social networks: II. Experimental results. *Soc. Netw.* 2 119–141. 10.1016/0378-8733(79)90002-9

[B15] Friedrich T. Vessey W. Schuelke M. J. Ruark G. A. Mumford M. D. (2009). A framework for understanding collective leadership: the selective utilization of leader and team expertise within networks. *Leadersh. Q.* 20 933–958. 10.1016/j.leaqua.2009.09.008

[B16] Gilstrap J. B. Collins B. J. (2012). The importance of being trustworthy: trust as a mediator of the relationship between leader behaviors and employee job satisfaction. *J. Leadersh. Organ. Stud.* 19 152–163. 10.1177/1548051811431827

[B17] Gøtzsche-Astrup O. (2018). The time for causal designs: review and evaluation of empirical support for mechanisms of political radicalisation. *Aggress. Violent Behav.* 39 90–99. 10.1016/j.avb.2018.02.003

[B18] Granovetter M. (1983). The strength of weak ties: a network theory revisited. *Sociol. Theory* 1 201–233.

[B19] Hafez M. Mullins C. (2015). The radicalization puzzle: a theoretical synthesis of empirical approaches to homegrown extremism. *Stud. Conflict Terrorism* 38 958–975. 10.1080/1057610x.2015.1051375

[B20] Hakimi N. Van Knippenberg D. Giessner S. (2010). Leader empowering behaviour: the leader’s perspective. *Br. J. Manage.* 21 701–716. 10.1111/j.1467-8551.2010.00703.x

[B21] Harari Y. N. (2016). *Homo Deus: A Brief History of Tomorrow.* New York, NY: Random House.

[B22] Hardin C. D. Higgins E. T. (1996). “Shared reality: how social verification makes the subjective objective,” in *Handbook of Motivation and Cognition. Handbook of Motivation and Cognition, The Interpersonal Context*, Vol. 3 eds Sorrentino R. M. Higgins E. T. (New York, NY: The Guilford Press), 28–84.

[B23] Harris S. (2005). *The End of Faith: Religion, Terror, and the Future of Reason.* New York, NY: WW Norton & Company.

[B24] Hennes E. P. Nam H. H. Stern C. Jost J. T. (2012). Not all ideologies are created equal: epistemic, existential, and relational needs predict system-justifying attitudes. *Soc. Cognit.* 30 669–688. 10.1521/soco.2012.30.6.669

[B25] Hoffman B. (2006). *Inside Terrorism.* New York, NY: Columbia University Press.

[B26] Hofstede G. Bond M. H. (1984). Hofstede’s culture dimensions: an independent validation using Rokeach’s value survey. *J. Cross-cultural Psychol.* 15 417–433. 10.1177/0022002184015004003

[B27] Hogg M. A. (2014). From uncertainty to extremism: social categorization and identity processes. *Curr. Direct. Psychol. Sci.* 23 338–342. 10.1177/0963721414540168

[B28] Irawanto D. W. (2009). An analysis of national culture and leadership practices in Indonesia. *J. Divers. Manage. Second Q.* 4 41–48. 10.1108/LHS-01-2016-0001 28128040

[B29] Jackson B. A. (2006). ‘Groups, networks, or movements: a command-and-control-driven approach to classifying terrorist organizations and its application to Al Qaeda’. *Stud. Conflict Terrorism* 29 241–262. 10.1080/10576100600564042

[B30] Jost J. Hunyady O. (2003). The psychology of system justification and the palliative function of ideology. *Eur. Rev. Soc. Psychol.* 13 111–153. 10.1080/10463280240000046

[B31] Jost J. T. Ledgerwood A. Hardin C. D. (2008a). Shared reality, system justification, and the relational basis of ideological beliefs. *Soc. Personal. Psychol. Compass* 2 171–186. 10.1111/j.1751-9004.2007.00056.x

[B32] Jost J. T. Nosek B. A. Gosling S. D. (2008b). Ideology: its resurgence in social, personality, and political psychology. *Perspect. Psychol. Sci.* 3 126–136. 10.1111/j.1745-6916.2008.00070.x 26158879

[B33] Koschade S. (2006). A social network analysis of Jemaah Islamiyah: the applications to counterterrorism and intelligence. *Stud. Conflict Terrorism* 29 559–575. 10.1080/10576100600798418

[B34] Koschade S. (2007). *The Internal Dynamic of Terrorist Cells: A Social Network Analysis of Terrorist Cells in An Autralian Context*. Dissertation, Queensland University of Technology, Brisbane, QLD.

[B35] Kruglanski A. W. Bélanger J. J. Gelfand M. Gunaratna R. Hettiarachchi M. Reinares F. (2013). Terrorism—A (self) love story: redirecting the significance quest can end violence. *Am. Psychol.* 68:559. 10.1037/a0032615 24128318

[B36] Kruglanski A. W. Bélanger J. J. Gunaratna R. (2019). *The Three Pillars of Radicalization: Needs, Narratives, and Networks.* Oxford, MS: Oxford University Press.

[B37] Kruglanski A. W. Gelfand M. J. Bélanger J. J. Sheveland A. Hetiarachchi M. Gunaratna R. (2014). The psychology of radicalization and deradicalization: how significance quest impacts violent extremism. *Political Psychol.* 35 69–93. 10.1111/pops.12163

[B38] Laqueur W. (2017). “The sociology of terrorism,” in *A History of Terrorism*, ed. White A. (London: Routledge), 79–132. 10.4324/9781315083483-3

[B39] Lin X. W. Che H. S. Leung K. (2009). The role of leader morality in the interaction effect of procedural justice and outcome favorability. *J. Appl. Soc. Psychol.* 39 1536–1561. 10.1111/j.1559-1816.2009.00494.x

[B40] Liu J. (2015). Globalizing indigenous psychology: an East Asian form of hierarchical relationalism with worldwide implications. *J. Theory Soc. Behav.* 45 82–94. 10.1111/jtsb.12058

[B41] Liu J. H. Li M. C. Yue X. (2010). “Chinese social identity and intergroup relations: the influence of benevolent authority,” in *Handbook of Chinese Psychology*, ed. Bond M.H. (Oxford: Oxford University Press), 579–597.

[B42] Magolda M. B. B. (2008). Three elements of self-authorship. *J. Coll. Student Dev.* 49 269–284. 10.1353/csd.0.0016

[B43] Matthew R. Shambaugh G. (2005). The limits of terrorism: a network perspective. *Int. Stud. Rev.* 7 617–627. 10.1111/j.1468-2486.2005.00536.x

[B44] Mayer R. C. Davis J. H. Schoorman F. D. (1995). An integrative model of organizational trust. *Acad. Manage. Rev.* 20 709–734. 10.5465/amr.1995.9508080335

[B45] McCauley C. Moskalenko S. (2011). *Friction: How Radicalization Happens to Them and Us.* New York, NY: Oxford University Press.

[B46] Milla M. N. (2005). Bias heuristik dalam proses penilaian dan pengambilan strategi terorisme. *J. Psikol. Indonesia* 1 9–21.

[B47] Milla M. N. (2010). *Mengapa Memilih Jalan Teror: Analisis Psikologis Pelaku Teror.* Yogyakarta: Gadjah Mada University Press.

[B48] Milla M. N. Faturochman Ancok D. (2013). The impact of leader–follower interactions on the radicalization of terrorists: a case study of the Bali bombers. *Asian J. Soc. Psychol.* 16 92–100. 10.1111/ajsp.12007

[B49] Milla M. N. Hudiyana J. (2019). The protecting role of cross-group friendship mediates the role of ideological quest for significance on commitment to radical group. *Psychol. Res. Urban Soc.* 2:98 10.7454/proust.v2i2.42

[B50] Milla M. N. Putra I. E. Umam A. N. (2019). ‘Stories from jihadists: significance, identity, and radicalization through the call for jihad’. *Peace Conflict: J. Peace Psychol.* 25 111–121. 10.1037/pac0000371

[B51] Milla M. N. Umam A. N. (2019). “Understanding intergroup contact on terrorist prisoners in Indonesia,” in *Learning From Violent Extremist Attacks: Behavioural Sciences Insights for Practitioners and Policymakers*, eds Khader M. Neo L. S. Tan J. Cheong D. D. Chin J. (Singapore: World Scientific).

[B52] Moghaddam F. M. (2005). The staircase to terrorism: a psychological exploration. *Am. Psychol.* 60:161. 10.1037/0003-066x.60.2.161 15740448

[B53] Newson M. Bortolini T. Buhrmester M. da Silva S. R. da Aquino J. N. Q. Whitehouse H. (2018). Brazil’s football warriors: social bonding and inter-group violence. *Evol. Hum. Behav.* 39 675–683. 10.1016/j.evolhumbehav.2018.06.010

[B54] Onraet E. Van Hiel A. Roets A. Cornelis I. (2011). The closed mind:‘Experience’and ‘cognition’aspects of openness to experience and need for closure as psychological bases for right-wing attitudes. *Eur. J. Personal.* 25 184–197. 10.1002/per.775

[B55] Passmore D. L. (2011). *Social Network Analysis: Theory and Applications.* Dehradun: D. L. Passmore.

[B56] Piazza J. A. (2017). Repression and terrorism: a cross-national empirical analysis of types of repression and domestic terrorism. *Terrorism Political Violence* 29 102–118. 10.1080/09546553.2014.994061

[B57] Pyszczynski T. Solomon S. Greenberg J. (2003). *In the Wake of 9/11: The Psychology of Terror.* Washington, D.C.: American Psychological Association.

[B58] Rahardjo S. (1994). Between two worlds: modern state and traditional society in Indonesia. *Law Soc. Rev.* 28 493–502.

[B59] Rajiani I. Jumbri I. A. (2011). A cultural ecology of new public management in Indonesia. *J. Admin. Sci.* 8 17–31. 10.1016/j.scitotenv.2019.01.077 30743965

[B60] Rausch C. C. (2015). Fundamentalism and terrorism. *J. Terrorism Res.* 6 28–35.

[B61] Ressler S. (2006). Social NETWORK ANALYSIS AS AN APPROACH TO COMBAT TERRORISM: PAST, PRESENT, AND FUTURE RESEArch. *Homeland Sec. Affairs* 2 1–10.

[B62] Rosemont H. Jr. (2015). *Against Individualism: A Confucian Rethinking of the Foundations of Morality, Politics, Family, and Religion.* Lanham, MD: Lexington Books.

[B63] Sageman M. (2004). *Understanding Terror Networks.* Philadelphia, PA: University of Pennsylvania Press.

[B64] Sageman M. (2011). *Leaderless jihad: Terror Networks in the Twenty-First Century.* Philadelphia, PA: University of Pennsylvania Press.

[B65] Sageman M. (2017). *Turning to Political Violence: The Emergence of Terrorism.* Philadelphia, PA: University of Pennsylvania Press.

[B66] Scott J. (1988). Social network analysis. *Sociology* 22 109–127.

[B67] Silber M. D. Bhatt A. Analysts S. I. (2007). *Radicalization in the West: The Homegrown Threat.* New York, NY: Police Department, 1–90.

[B68] Stern J. (2003). *Terror in the Name of God: Why Religious Militants Kill.* New York, NY: Harper Collin Publishers.

[B69] Triandis H. C. Bontempo R. Betancourt H. Bond M. Leung K. Brenes A. (1986). The measurement of the etic aspects of individualism and collectivism across cultures. *Austr. J. Psychol.* 38 257–267. 10.1080/00049538608259013

[B70] Webber D. Babush M. Schori-Eyal N. Vazeou-Nieuwenhuis A. Hettiarachchi M. Bélanger J. J. (2018). The road to extremism: field and experimental evidence that significance loss-induced need for closure fosters radicalization. *J. Pers. Soc. Psychol.* 114:270. 10.1037/pspi0000111 28872332

[B71] Wheatley M. J. (2007). Leadership of self-organized networks, lesson from the war on terror. *Peform. Inform. Improve. Quart.* 20 59–66. 10.4278/ajhp.120912-QUAN-443 23971523 PMC4752846

[B72] Whitehouse H. Yustisia W. Putra I. E. Kavanagh C. Rufaedah A. (2019). The role of religious fundamentalism and tightness-looseness in promoting collective narcissism and extreme group behavior. *Psychol. Religion Spirituality*

[B73] Wiktorowicz Q. (2005). *Radical Islam rising: Muslim extremism in the West. Oxford, United Kingdom.* Lanham, MD: Rowman & Littlefield.

[B74] Yamagishi T. Cook K. Watabe M. (1998). Uncertainty, trust, and commitment formation in the United States and Japan. *Am. J. Sociol.* 104 165–194. 10.1086/210005

[B75] Yuki M. (2003). Intergroup comparison versus intragroup relationships: a cross-cultural examination of social identity theory in north american and east asian cultural contexts. *Soc. Psychol. Quart.* 66 166–183.

