# Supplementary material for: Is the Role of Ideologists Central in Terrorist Networks? A Social Network Analysis of Indonesian Terrorist Groups
Source: Front Psychol. 2020 Mar 3;11:333. doi: 10.3389/fpsyg.2020.00333 (PMC7063091; doi:10.3389/fpsyg.2020.00333)
Supplement: Supplementary file 1 [file Data_Sheet_1.PDF]

Appendix A: Verbatim Responses

Table 1. Verbatim response for the qualitative analysis

#	Initials (Roles)	Style of Trustworthiness (Mayer et al., 1995)	The Needs Provided to Members (Jost et al., 2008)	Verbatim example
1.	IL1 (Ideological leader)	Ability – trustworthy because of the profound knowledge in religion	Epistemic needs – satisfying the needs of order and certainty	“The person who died in the field of jihad, is automatically become martyr in Allah’s view... at that moment, all sins are forgiven and the person will go directly to heaven, where they will be granted the most noble place by God. The person can bring 72 members of his family with him…”<br>(IL1 lecture in Krobokan Prison, Bali, Indonesia)
2.	IL2 (Ideological leader)	Ability – trustworthy because of the profound knowledge in religion	Epistemic needs – satisfying the needs of order and certainty	“IL2 said to me, when I was about to go to Poso, that it was not my time yet to join the fight because my level of religiousness was not there yet.”<br>(Interview with WY, follower of IL2, Police Headquarters, Depok, Indonesia)
3.	IL3 (Ideological leader)	Ability – trustworthy because of the profound knowledge in religion	Epistemic needs – satisfying the needs of order and certainty	“It is clear that whatever we experience in the way of Allah, is the true moral deeds. If we are killed, then we will gain a special place in Allah’s eyes. If we are imprisoned, then we will gain intimate time with Allah whilst keeping us away from sins. Everything that we experience in the way of Allah, even in the form of difficulties, misery, and pain, or in our attitude that makes the infidels furious towards us, can be considered as good deeds.”<br>(Quote by IL3, from his book entitled “Pearl Behind Prison: Reflection for the Faithful”)
4.	OL1 (Operational leader)	Benevolence – trustworthy because the leader provide resource and material needs	Existential needs – satisfying the needs to reduce stress	“Worldly materials and luxury goods (facilities and goods from the non-Muslims) cause the death of our spirit, weakening the Mujahideen mentality, so that we are weak in the face of enemy tricks, afraid to face the test on this road and leave the way of holy jihad. Some even turned out to be the traitors of jihad and mujahideen, weakening the Muslims and helping the enemies of Allah.”<br>(The sayings of OL1, retrieved from http://www.7ihadmedia.wordpress.com)

5.	OL2 (Operational leader)	Integrity – trustworthy because the leader value the group norms and inspire discipline	Relational needs – satisfying the need for devotion	“OL2, was the one who gave me a special place for experiments (explosive laboratory). I was basically asked to prepare explosives, where all my needs and other materials were provided. I was asked to make bombs, until eventually I was dubbed as The Master... He said that this is for Ambon (Poso bombing), so I just came along... It’s always like that with OL2... if I disobey him, then he will not go easy on me... "<br>(Interview with WF, a bomb maker of Al Qaeda Indonesia group and follower of OL2)
6.	OL3 (Operational leader)	Benevolence – trustworthy because the leader inspire loyalty as a good friend	Relational needs – satisfying the needs of social relationship	"I did plan to go for jihad. At that time, I was still in the Philippines, but OL3 contacted me to help with the Bali Bombing. I was actually quite doubtful... because Bali was not a conflict zone. But you know, OL3 is my friend. So I just comply... similar to when I was training in Aceh for the jihad to Palestine. OL3 suddenly contacted me, and he asked me to help... So I finally got arrested... well he is my friend, I can never refuse... "<br>(Interview with UP, Bali Bomb I Assembler and follower of OL3)
7.	OL4 (Operational leader)	Integrity – trustworthy because the leader value the group norms and inspire discipline	Relational needs – satisfying the need for devotion	"I actually didn't want to go to Bali at that time, because I was working as a teacher ... but OL4 insisted on telling me to go. So I said, yeah, and I obeyed him, because OL4 was the leader even though he is extremely ill-tempered..."<br>(AI interview, JI member Bali I)
8.	OL5 (Operational leader)	Benevolence – trustworthy because the leader provide paternalistic figure	Relational needs – satisfying the need for devotion	“Once, I was offered a meeting with someone named WW... turns out, it was the alias of OL5. OL5 was the fugitive at that time ... this the person was very fatherly, even gentle sometimes... Some of my friends who intend to join the jihad, they join through him....”<br>(Interview with YS, follower of OL5)
9.	OL6 (Operational leader)	Integrity – trustworthy because the leader value the group norms and inspire discipline	Relational needs – satisfying the need for devotion	“He has long been known by the people (as a leader figure) ... he once led a group of thugs in Poso before he led a jihad in the forest. His followers were many... the tough ones… his wife also joined him in the forest. I myself, just help, as a courier ... help with the logistics, in the kitchen .....”<br>(Interview with RK, follower OL6).

Appendix B: Short Bio of the Leaders

Ideological Leaders

IL1 was the fifth child from a family with eight children. Three of his brothers were convicts of the Bali Bombing. He was formally educated in the Islamic Boarding School, where he attained a *mu'alimin* degree (certified religious teacher). IL1 then got a job as a teacher, before he eventually went to a jihad in Afghanistan. After his return from the Afghanistan by the year of 1991, he married a Malaysian woman who were loyal to him and share his religious views and live in Malaysia. IL1 was known as a figure with profound religious knowledge and sheer intellect. He was a leader of an Islamic Boarding School in Malaysia before the school was closed by Malaysian governments for the accusation of violent extremist agenda. He was then a fugitive so he had to move to Indonesia along with his wife and children by the year of 2002. He was involved in the first Bali Bombing terrorist network, where he served as preacher and Imam (Islamic religious leader). He was eventually captured by the police, then sentenced to death by the year of 2003. During his time in prison, he perceived his imprisonment as a spiritual time where he can be alone with god. He was executed by Indonesian government in 2008.

IL2 had studied at Gadjah Mada University (UGM), a highly prestigious Indonesian university, but was not finished. His image as a cold-blooded terrorist in the public eye was in stark contrast with how his neighbours saw him. His neighbours perceived him as a warm, kind, and nice person. He possessed good communication and interpersonal skills, which is the reason why his sayings were easily obeyed by the followers. He was very fluent in explaining and describing the fundamental basis of Islamic laws, and can easily provide the basis of justification for violent actions. He was often called ustadz (Islamic Imam) and often preached in different cities in Indonesia. He joined the Afghanistan military camp in 1987 and was

quickly assigned as a mentor for his juniors, who joined after him. After this, IL2 was assigned as an ideological leader for Jama’ah Islamiyah group, which operated in Indonesia and the Philippines. He was a very charismatic and respected leader, a person whom followers trust him unquestioningly. He has been released from Indonesian prison and known as one of the former terrorists that cooperatively with the government in deradicalization program.

IL3 was the leader of the Ansharut Daulah Jamaat (JAD), which was inspired by ISIS. IL3 is a preacher who always emphasise the theme of *Tawheed* (philosophy of Islamic god) in his sermon. He was a graduate from LPIA, a reputable Islamic school in Jakarta, Indonesia. He was also very fluent in the Arabic language. His teachings often emphasize the purity of Islam and the concept of taghut (everything that is worship other than Allah). His speeches often inspire young people to be involved in terrorist activity. IL3 mention is associated with many of the terrorist actions in Indonesia. Some of these terrorist act was inspired by the speech from IL3. Among the terrorists who were inspired by the IL3 speech were they who involved in Thamrin attack, Jakarta, Indonesia and the bombing of Oikumene church, Samarinda, Indonesia in 2016. He was dubbed as the pioneer of ISIS movement in Indonesia and known as a preacher who giving strong call to fight the Indonesian government which was considered a taghut for not implementing Islamic sharia.

Operational Leaders

OL1 finished his undergraduate degree in Qur’an seminary. He was quite experienced in the activity of jihadi movement, in which he was assigned as the commander of *Mujaheedin* army and has been successful in sabotaging the police force’s weaponry warehouse in Ambon, Indonesia by the year of 2000. He was also known as a person who has a very large number of social acquaintances and quite influential in his own social circles. He was also known to actively recruit followers (usually young males) to his own jihadi group, which was based in

Ambon and Poso, Indonesia. By the time this paper is being written, OL1 is still in custody within Nusakambangan prison, Indonesia.

OL2 was an important figure who formed an organization named Al-Qaeda Indonesia, inspired by Bin Laden's Al-Qaeda movement. Al-Qaeda Indonesia was a network consisted of several distinct terrorist groups. The central operation was in Poso, Indonesia where the constant civil war between Muslims and Christians took place. According to *ketua RT* (the chief in OL2's neighbor), OL2 was rarely seen in the activities within the neighborhood From time to time, he withdrew from the activities involving the neighbors. He then moved to Poso, Indonesia where he preached the teachings of Abu Bakar Ba'asyir (a central figure in Indonesian and Malaysian violent extremist movement). He often preached in front of young males and he often recruit the members from this specific demography either. He was very skilled in making explosives and inspire young people to join his movement. He is currently being detained in Cibinong prison, Indonesia.

OL3 was known in his hometown, Pemalang, Central Java, Indonesia as an extroverted person and quite outgoing as well. OL3 and his family was known to be educated and can be classified as in the middle-upper economic status. His wife was admitted to the school of medicine in one of the most prestigious universities in Java, Indonesia to pursue a degree as a medical doctor. He was also dubbed as "the genius" by the jihadist networks because he was very skilled in electronic devices. Because of his reputation as 'the smart one', people in the terrorist networks respected him and looked up to him. The people who were close to him said that OL3 was very confident, rarely doubtful, and can be aggressive sometimes. OL3 was a senior member of *Jama'ah Islamiyah*, one of the largest terrorist groups in Southeast Asia. He assisted in training the Muslim rebellious army, especially in making explosives.

OL4 is a ninth child from a family with 11 children. Ever since he was a teenager, he lived only with his mother because his father left him to live with another woman. He was born

in January 14th, 1971. By the time he finished high school, he admitted that he found a spiritual enlightenment through the short *pesantren* (Islamic boarding school) activity. Since then, he isolated himself from his peers and social networks while seeking only social interactions with those whom he saw as deeply religious. These new networks eventually brought him to pursue identity as jihadist in Afghanistan. By the time he was in Afghanistan (1991-1993), he went through an intense military training to prepare him in the holy battle of jihad. OL4 was also very skilled in computer programming. After he returned, he was involved in several terrorism activities, most notably as the orchestrator of the first Bali Bombing where there were more than 200 people killed. In 2009, he was executed by Indonesian government.

OL5 was born in Johor Bahru, Malaysia by the year of 1968. He took an undergraduate degree in accountancy, Universiti Teknologi Malaysia (a highly prestigious university in Malaysia). According to his past colleague, AB, OL5 was such a humble person. He was quite modest, yet courageous. His attitude was often very consistent. AB said that OL5 chose the path to terror because he followed the *fatwa* (preaching) from Osama bin Laden, supreme leader of Al-Qaeda, who is a well-known figure in terrorist movement. More specifically, he followed the teaching that United States was the nemesis of Islam, since United States has oppressed Muslim societies such as Irak and Afghanistan. He joined the *Jama'ah Islamiyah* movement and orchestrated at least four major bombings in Indonesia, such as J.W. Marriot bombing in 2003, Australian Embassy bombing in 2004, Bali bombing II in 2005, and Mega Kuningan bombing in 2009. Shortly after the bombing in 2009, he died of a shot by the Indonesian police officer.

OL6 was the leader of *Mujahidin Indonesia Timur* (MIT) group, a terrorist network which was inspired by Islamic State of Irak and Syria (ISIS). OL6 was born in Poso, Indonesia by the year of 1967. A constant civil war between Muslims and Christians in Poso has transformed his life. His peers often consisted of those who were already became fighter in

Poso interreligious conflict, and his relationship with them led OL6 to become more interested in religious teachings. He sold religious books, and he often read the books that he sold. He then participated in Aceh Military Training, which, in turn led him to establish his own terrorist network. His name was known to the public when he attempted to assault the police officers in 2011. He was known as a charismatic leader in the eyes of his followers. He is well respected because of his status as commander of Poso military training. His followers completely obeyed him because of these two factors.
